# Kinetics and Energetics of Phylloquinone Reduction in Photosystem I: Insight From Modeling of the Site Directed Mutants

**DOI:** 10.3389/fpls.2019.00852

**Published:** 2019-07-02

**Authors:** Stefano Santabarbara, Anna Paola Casazza

**Affiliations:** ^1^Centre for Fundamental Research in Photosynthesis, Vergiate, Italy; ^2^Photosynthesis Research Unit, Centro Studi sulla Biologia Cellulare e Molecolare delle Piante, Milan, Italy; ^3^Istituto di Biologia e Biotecnologia Agraria, Consiglio Nazionale delle Ricerche, Milan, Italy

**Keywords:** photosystem I, electron transfer, phylloquinone, kinetic modeling, bioenergetics, redox tuning

## Abstract

Two phylloquinone molecules (*A*_1_), one being predominantly coordinated by PsaA subunit residues (*A*_1A_) the other by those of PsaB (*A*_1B_), act as intermediates in the two parallel electron transfer chains of Photosystem I. The oxidation kinetics of the two phyllosemiquinones by the iron-sulfur cluster F_X_ differ by approximately one order of magnitude, with $A_{1\text{A}}^{-}$ being oxidized in about 200 ns and $A_{1\text{B}}^{-}$ in about 20 ns. These differences are generally explained in terms of asymmetries in the driving force for F_X_ reduction on the two electron transfer chains. Site directed mutations of conserved amino acids composing the *A*_1_ binding site have been engineered on both reaction center subunits, and proved to affect selectively the oxidation lifetime of either $A_{1\text{A}}^{-}$, for PsaA mutants, or $A_{1\text{B}}^{-}$, for PsaB mutants. The mutation effects are here critically reviewed, also by novel modeling simulations employing the tunneling formalism to estimate the electron transfer rates. Three main classes of mutation effects are in particular addressed: (i) those leading to an acceleration, (ii) those leading to a moderated slowing (~5-folds), and (iii) those leading to a severe slowing (>20-folds) of the kinetics. The effect of specific amino acid perturbations contributing to the poising of the phylloquinones redox potential and, in turn, to PSI functionality, is discussed.

## Overview of Electron Transfer IN Photosystem I

**Figure 1:**
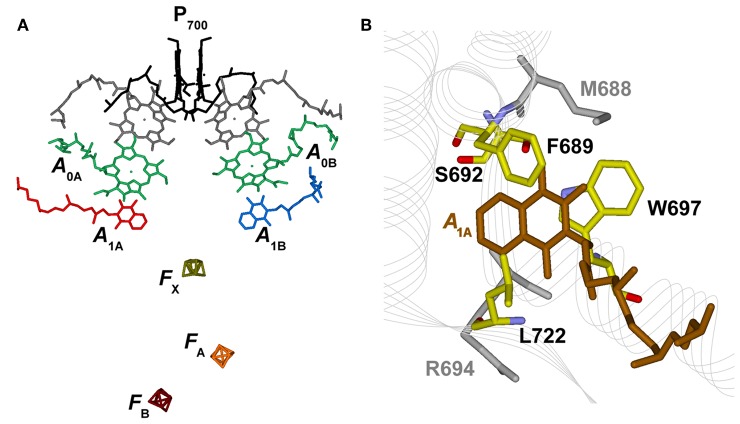
**(A)** Structural organization of the co-factors composing the electron transfer chains in Photosystem I from Jordan et al. (2001), PDB file # 1JB0. Shown are: the terminal electron donor (P_700_) considered as Chl *a*/Chl *a*′ hetero-dimer (black); the so-called accessory Chl *a* are unlabeled (gray); the primary Chl *a* electron acceptors A_0(A/B)_ (green); the phylloquinones A_1A_ (red) and A_1B_ (blue) and the terminal 4Fe-4S clusters, F_X_ (gold), F_A_ (orange), and F_B_ (burgundy). **(B)** Phylloquinone binding niche of the PsaA reaction center subunit. The phylloquinone molecule (A_1A_) is shown in brown. The atom color-coded (C: yellow, O: red, N: blue) side chains represent the principal protein residues interacting with the redox moiety. Mutations of these specific residues are discussed in the text. Side chains shown in gray also have a role in controlling ET properties but will not be specifically discussed here.

Photosystem I (PSI) is a key component of both the linear and the cyclic electron transport chains of oxygenic photosynthesis. Electron transfer (ET) reactions take place in a protein-cofactor super-complex known as the core, which is overall well conserved throughout evolution. The core complex of PSI is composed by over 20 protein subunits, the exact number being species-specific. Most of the cofactors involved both in light harvesting and ET reactions are bound by the heterodimer composed of the PsaA and PsaB protein subunits (Jordan et al., 2001; Qin et al., 2015; Mazor et al., 2017). The most notable exception are two iron-sulfur clusters, named *F*_A_ and *F*_B_, which are bound by the PsaC subunit instead. Structural studies indicate that the cofactors involved in ET reactions are related to a C_2_ (mirror) symmetry (Figure 1) with respect to an axis perpendicular to the putative membrane plane (Jordan et al., 2001; Qin et al., 2015; Mazor et al., 2017), which is a common structural motif of photosynthetic reaction centers. However, a large body of evidences has been gathered leading to the conclusion that, differently from the other well-characterized systems such as the purple bacteria RC and Photosystem II, in PSI both cofactors chains are ET active (Joliot and Joliot, 1999; Guergova-Kuras et al., 2001; Muhiuddin et al., 2001; Santabarbara et al., 2005a, 2010a; Rappaport et al., 2006; Redding and van der Est, 2006; Srinivasan and Golbeck, 2009; Makita and Hastings, 2016).

Electron transfer reactions are initiated from the lowest singlet excited state of the chromophores composing the photochemically active center, giving rise to the population, via one or more intermediate steps, of a radical pair consisting of a chlorophyll (Chl) cation, residing on the metastable electron donor ($P_{700}^{+}$), and a phyllosemiquinone anion ($A_{1}^{-}$). Two specific radical pairs, attributed to $\left[P_{700}^{+}A_{\text{1A}}^{-}\right]$ and $\left[P_{700}^{+}A_{\text{1B}}^{-}\right]$ (the subscript refers to the subunit harboring the phylloquinone), were identified in PSI, demonstrating the photochemical functionality of both ET branches (Poluektov et al., 2005; Santabarbara et al., 2005b, 2006, 2010c; Berthold et al., 2012). Successive ET reactions involve the cascade oxidation of $A_{1}^{-}$ initially by the PsaA:PsaB coordinated cluster *F*_X_, and successively by the PsaC-coordinated clusters *F*_A_ and *F*_B_, that are oxidized by diffusible electron carries, the most common of which is ferredoxin. Noticeably, *F*_X_ represents a point of convergence of the two active ET branches, as it is shared by both, and so are the terminal acceptors *F*_A_ and *F*_B_ (Figure 1).

In place of a remarkable structural symmetry of the active ET chains, they also show clear functional differences, the most evident being the approximately one order of magnitude slower oxidation lifetime of $A_{1\text{A}}^{-}$ with respect to $A_{1\text{B}}^{-}$ at room temperature. These two reactions are characterized by apparent lifetimes in the 200–350 and 5–30 ns intervals, respectively (e.g., Brettel, 1997; Santabarbara et al., 2005a, 2010a; Rappaport et al., 2006; Srinivasan and Golbeck, 2009; Makita and Hastings, 2016 for reviews). The assignment of these oxidation components to ET events involving either $A_{1\text{A}}^{-}$ or $A_{1\text{B}}^{-}$ was based on the study of site-selective mutants of the phylloquinone binding niches of either the PsaA or the PsaB subunit, leading to specific alterations of either the “slow” (~250 ns) or the “fast” (~20 ns) lifetime (Guergova-Kuras et al., 2001; Xu et al., 2003; Byrdin et al., 2006; Santabarbara et al., 2008, 2010b, 2015; Srinivasan et al., 2011). Moreover, not only the apparent oxidation lifetimes of $A_{1\text{A}}^{-}$ and $A_{1\text{B}}^{-}$ differ significantly, but they also have distinct temperature dependences, with ~250 ns showing a significant thermal activation (100–130 meV, Schlodder et al., 1998; Agalarov and Brettel, 2003; Santabarbara et al., 2009) and the ~20 ns being only weakly temperature dependent (7–43 meV, Agalarov and Brettel, 2003; Santabarbara et al., 2009). The complexity of $A_{1}^{-}$ oxidation at physiological temperature is also highlighted by the presence of an additional kinetic phase, sometimes referred to as “intermediate,” having lifetimes in the 150–180 ns range. This component was first observed in investigations of the reaction temperature dependence (Agalarov and Brettel, 2003) and successively in mutants of the PsaA subunit affecting the $A_{1\text{A}}^{}$ binding niche (Byrdin et al., 2006; Santabarbara et al., 2008). It has been tentatively assigned to ET involving the 4Fe-4S clusters. The above discussed differences in the kinetic behavior of $A_{1\text{A}}^{-}$ and $A_{1\text{B}}^{-}$ represent a nice example of the specific involvement of protein binding and coordination in tuning the redox properties of cofactors active in ET reactions. This is a well-recognized phenomenon which is also apparent for the 4Fe-4S clusters bound to PSI, that although being chemically indistinguishable, display differences in the redox potentials well exceeding 150 mV, based on titrations, which pose *F*_A_ and *F*_B_ in the –(500–550) mV range (Ke et al., 1973, 1977; Evans et al., 1974; Lozier and Butler, 1974; Heathcote et al., 1978; Evans and Heathcote, 1980; Nugent et al., 1981) and F_X_ in the –(670–730) mV interval [(Ke et al., 1977; Chamorowsky and Cammack, 1982; Parrett et al., 1989) and the detailed discussion by Brettel (1997) on the limits of these estimations]. On the other hand, direct titration of $A_{1}^{}$ has proven to be very difficult because of its very negative redox midpoint potential. Henceforth, to acquire information on the redox properties of $A_{1\text{A}}^{}$ and $A_{1\text{B}}^{}$, alternative approaches based either on theoretical chemical methods (Ishikita and Knapp, 2003; Karyagina et al., 2007; Ptushenko et al., 2008; Kawashima and Ishikita, 2017) or on modeling of the ET reactions kinetics in the framework of non-adiabatic ET tunneling theory (Santabarbara et al., 2005a; Moser and Dutton, 2006; Makita et al., 2015; Santabarbara and Zucchelli, 2016; Cherepanov et al., 2017; Makita and Hastings, 2017; Milanovsky et al., 2017) have been adopted.

Still, the predictions from these two approaches do not generally agree, despite showing some common traits, the most relevant of which being that the estimated redox potential (*E*^0^) of the $A_{1\text{B}}^{-}/A_{1\text{B}}^{}$ pair is more negative than that of $A_{1\text{A}}^{-}/A_{1\text{A}}^{}$. Structure-based theoretical calculations tend to predict a difference in potential between the two phylloquinone (PhQ) molecules exceeding 100 mV, whereas kinetic modeling predicts the difference to be in the order to 40–60 mV.

Although the latter approach appears more indirect, it is constrained to satisfactorily reproduce the ET kinetics, at least to a semi-quantitative level. Comparing different energetic landscapes, it was concluded that a scenario involving a weakly endergonic oxidation of $A_{1\text{A}}^{-}$ (standard free energy difference, $\Delta G_{{\text{A}}_{\text{1A}}}^{\text{0}}$ of +10 meV) and a larger ($\Delta G_{{\text{A}}_{\text{1A}}}^{\text{0}}$ = −50 meV) exergonic oxidation of $A_{1\text{B}}^{-}$ provided a good description of both the room temperature kinetics as well as their temperature dependences (Santabarbara and Zucchelli, 2016). Scenarios involving largely exergonic oxidation of both $A_{1\text{A}}^{-}$ and $A_{1\text{B}}^{-}$ (e.g., $\Delta G_{{\text{A}}_{\text{1A}}}^{\text{0}}$ = −86 meV and $\Delta G_{{\text{A}}_{\text{1B}}}^{\text{0}}$ = −259 meV from Ptushenko et al. (2008); note the subscript indicates the electron donor) were also shown to be compatible with the experimental results, although they required a larger value of the (total) reorganization energy and concomitantly predicted a rather slow oxidation of reduced *F*_X_ (Santabarbara and Zucchelli, 2016). At the same time Makita and Hastings (2017), based on analogous kinetic approaches but also considering the effect of replacing the naturally occurring PhQ with exogenous redox moieties having inherently different redox midpoint potentials, obtained results comparable with the weak driving force scenario, but with differences in the best-describing driving force estimation as $\Delta G_{{\text{A}}_{\text{1A}}}^{\text{0}}$ = 45 meV and $\Delta G_{{\text{A}}_{\text{1B}}}^{\text{0}}$ = −10 meV (Makita et al., 2015; Makita and Hastings, 2017). Moreover, these authors showed that considering the effect of different ET processes, for instance the forward oxidation kinetics and the charge recombination reaction between the ET intermediates (i.e., reduced *F*_A_ and *F*_B_) and the terminal electron donor $P_{700}^{+}$, imposed some critical constrain to the model parameters other than the Gibbs free energy (Makita and Hastings, 2017). In their modeling framework, large reorganization energies such as those necessary to describe forward ET in the “largely exergonic” scenario, did not lead to acceptable descriptions of the recombination kinetics. Moreover, their arguments imply that all ET reactions downstream of $A_{1\text{A}}^{-}$ and $A_{1\text{B}}^{-}$ need to be considered in order to properly account for the charge recombination processes.

As highlighted in a recent critical review (Santabarbara et al., 2019) specific choices of parameter sets can have significant impact on the back estimation of $\Delta G_{}^{\text{0}}$ (and therefore on the $E_{}^{0}$) from kinetic modeling. Yet, a consensus energetic scenario was retrieved in which $A_{1\text{A}}^{-}$ oxidation fell in the weakly exergonic to weakly endergonic picture (−5 < $\Delta G_{{\text{A}}_{\text{1A}}}^{\text{0}}$ <35 meV) and $A_{1\text{B}}^{-}$ oxidation was generally exergonic, with the associated driving force being larger than −10 meV (−50 < $\Delta G_{{\text{A}}_{\text{1B}}}^{\text{0}}$ < -10 meV).

To further resume the potentiality of theory-bound kinetic modeling in uncovering the ET energetics in PSI, in the following paragraphs the basic principles of ET rates description and of kinetic simulations will be briefly introduced. This will be done in association to a semi-quantitative modeling of both the energetics and the kinetics of $A_{1}^{-}$ oxidation in wild-type PSI reaction center. The effect of mutations affecting the PhQ binding sites, in particular at the level of the PsaA subunit, will then be discussed, and, in this context, kinetic simulations will also be presented for the mutant scenarios.

## Basic Principles of Electron Transfer Theory

The ET *rates* between a donor-acceptor cofactor pair can be described by the following equation, which represents a simplified form of the expression originally derived by Hopfield (1974); Devault (1980), but considering a single (mean) nuclear mode coupled to the ET event:

(1) $$\begin{array}{l} \;k_{D\to A}=\frac{2\pi }{\hbar }{\left|H_{DA}\right|}^{2}\frac{1}{\sqrt{2\pi \sigma^{2}}}e^{-\frac{{\left(\Delta G_{{}^{DA}}^{0}+\lambda \right)}^{2}}{2\sigma^{2}}}\\ \;\sigma^{2}=\lambda \hbar \bar{\omega }\coth\frac{\hbar \bar{\omega }}{2k_{B}T}\\ {\left|H_{DA}\right|}^{2}={\left|H_{0}\right|}^{2}e^{-\beta \left(X_{DA}-3.6\right)} \end{array}$$

In Equation (1), ${\left|H_{DA}\right|}^{2}$ is the electronic coupling term described by ${\left|H_{0}\right|}^{2}$, its maximal value at wavefunction overlap, β, a damping term associated to the probability of tunneling the potential barrier and X_*DA*_, the edge-to-edge cofactor distance. The 3.6 factor is a correction for the van der Waals radii. λ is the (total) reorganization energy, $\bar{\omega }$ is the angular frequency of the mean coupled nuclear mode, ℏ is the Dirac constant, *k*_*B*_ is the Boltzman constant and *T* is the temperature. When $\hbar \bar{\omega }$ < < *k*_*B*_*T* the expression simplifies to: $\sigma_{}^{2}=2\lambda k_{B}T$, yielding the semi-classical one derived by Marcus (Marcus and Sutin, 1985). An expression which considers multiple modes and specific electron-phonon coupling and that provides a more accurate description of temperature dependence over a broad temperature range was derived by Jortner (1976). However, employing Jortner formalism leads inevitably to a large increase in the number of parameters to be considered, and most of the deviations with respect to Equation 1 are predicted at low and intermediate temperatures. Hence, the Hopfield-Marcus expressions represent a good approximation, at least at physiological temperatures. Noticeably, Equation 1 has been parameterised by Moser and Dutton (1992) and Moser and Dutton (1996) in a linearised form on logarithmic scale that at room temperature is: $\log\left(k_{D\to A}\right)=13-0.6\cdot \left(X_{DA}-3.6\right)-3.1\frac{{\left(\Delta G_{DA}^{0}+\lambda \right)}^{2}}{\lambda }$. The parameterised values are equivalent to ${\left|H_{0}\right|}^{2}$~10^−3^ eV^2^, β = 1.38 Å^−1^ and $\hbar \bar{\omega }$ = 56 meV in Equation 1. Similar values will be adopted in the forthcoming calculations (${\left|H_{0}\right|}^{2}$ = 1.3 10^−3^ eV^2^, β = 1.34 Å^−1^) but $\hbar \bar{\omega }$ will instead be taken from the analysis of the temperature dependences of these electron transfer reactions (Mula et al., 2012), i.e., $\hbar {\bar{\omega }}_{A_{1A}\to F_{X}}$ = 21 meV and $\hbar {\bar{\omega }}_{A_{1B}\to F_{X}}$ = 47 meV. The values of $\Delta G_{DA}^{0}$ and λ will be discussed below (see Table 1 for compilation of the input parameters).

**Table 1:** Principal parameters inputs employed in the simulations of ET in wild-type PSI.

	**Common**		**Weak driving force**			**Large driving force**		
**Reaction**	***X*_*DA*_ (Å)**	**$\bar{\omega }$ (eV)**	**λ_*tot*_ (eV)**	**$\Delta G_{DA}^{0}$ (eV)**	***k*_*D*→*A*_ (ns^−1^)**	**λ_*tot*_ (eV)**	**$\Delta G_{DA}^{0}$ (eV)**	***k*_*D*→*A*_ (ns^−1^)**
$A_{1A}^{-}\to F_{X}$	9.1	0.021	0.700	0.010	0.0180	1.0	−0.086	0.00508
$A_{1B}^{-}\to F_{X}$	9.0	0.047	0.700	−0.050	0.152	1.0	−0.259	0.195
$F_{X}^{-}\to F_{A}$	11.6	0.019	0.700	−0.160	0.0105	1.0	−0.16	0.000556
$F_{A}^{-}\to F_{B}$	9.5	0.019	0.825	0.025	0.00189	1.0	0.025	0.000324

*Summary of the parameters utilized for the simulations of forward electron transfer in wild-type PSI considering two possible energetic scenarios. Other parameters common to all reactions (and to both energetic scenarios) are: the electronic coupling matrix element, ${\left|H_{0}\right|}^{2}$ = 1.3 10^−3^ eV^2^, the barrier damping factor,β = 1.34 Å^−1^, and the temperature, T = 290 K (see Equation 1 in the text). For all the recombination reactions λ = 0.825 eV was used in the weak driving force scenario, whereas λ = 1.0 eV was employed in the large driving force framework*.

From Equation (1) (and equivalently from the parameterised “ET ruler” expression) the maximal rate of ET (for any given ${\left|H_{DA}\right|}^{2}$) is obtained when $\Delta G_{DA}^{0}=-\lambda$. Under these conditions the temperature dependence of the reaction is minimal. The absolute maximal rate is attained not only when $\Delta G_{DA}^{0}=-\lambda$ but also when ${\left|H_{DA}\right|}^{2}$ = ${\left|H_{0}\right|}^{2}$, i.e., at minimal distance.

## Kinetic Simulations

**Figure 2:**
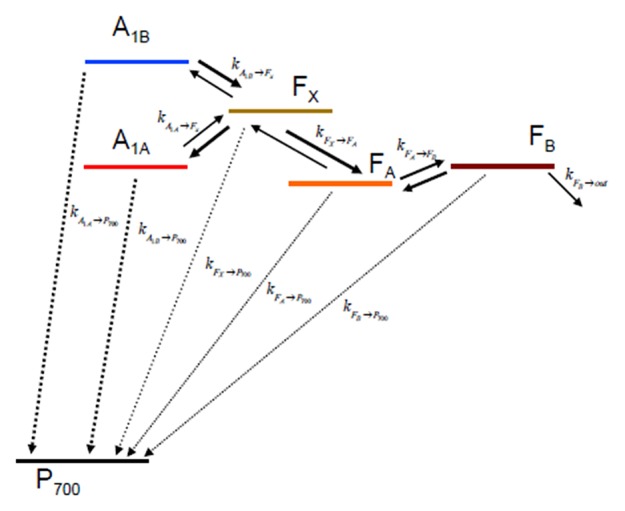
Kinetic scheme describing the electron transfer reactions downstream of *A*_1_ and the recombination paths to $P_{700}^{+}$ considered in the simulations.

Once the ET rates are computed (Equation 1) they can be inserted in an appropriate kinetic model which depends on the reaction scheme considered (Figure 2). In case ET involves only one-electron oxido-reductions, like in the case of PSI, the temporal evolution of the ET cofactor populations can be described by a system of coupled ordinary differential equations having, in compact matrix notation, the general form: **D**(*t*) = **K**_*i*_·**D**(*t*). **D**(*t*) is a vector describing the temporal evolution of the redox intermediate populations and **D**(*t*) is its first derivative with respect to time. Both vectors have the same dimension equalling the number of ET steps in the redox chain, *i*. **K**_*i*_ is a square matrix containing the molecular rate constants between couples of donors-acceptors as its elements and is often called the *rate matrix*. Elements on the diagonal include the depopulation of redox intermediates whereas the off-diagonal ones are the formation/population rates. This system has general solutions of the form: $\text{D}\left(t\right)=\overset{i}{\underset{J=1}{\sum }}c_{j}{\text{V}}_{j}e^{\zeta_{j}t}$, where ζ_*j*_ and **V**_*j*_ are, the eigenvalues and the eigenvectors, respectively, necessary to diagonalise **K**_*i*_. The eigenvalues are univocally determined and relates to the experimentally observed lifetimes ($\tau_{j,obs}^{}$) by the simple relation $\tau_{j,obs}^{}=-\zeta_{j}^{-1}$. It is very commonly assumed that the inverse of the experimentally determined lifetimes ($\tau_{j,obs}^{-1}$) describes to a good approximation the ET rate constants. This is however reasonable only as long as the off-diagonal terms of **K**_*i*_ are much smaller than the diagonal ones. In general the eigenvalues, and therefore the observed lifetimes, will depend on all the rate constants coupling the different intermediates. In order to unambiguously define the pre-exponential amplitude, scaling constants (*c*_*j*_) weighting the eigenvectors of **K**_*i*_ need to be determined. This requires solving a boundary problem, which is most commonly determined by the initial conditions (e.g., the concentration of the intermediates at *t* → 0). As discussed for the eigenvalues/lifetime relation, also the eigenvectors elements (amplitudes) cannot be directly associated to specific ET events, because their values will depend on all of the rate constant composing the **K**_*i*_ matrix, as well as on the boundary conditions (see Santabarbara et al., 2014 for further discussion). The statistical utilization of the two active ET chains in PSI, which ideally defines the boundary, is still somewhat debated and might be dependent on the class of organisms considered. Whereas, in model green algae the two branches appear to be almost equally active in ET reactions (e.g., Guergova-Kuras et al., 2001; Santabarbara et al., 2005a,b, 2015), more asymmetric utilization, in favor of the PsaA branch (up to 0.8:0.2), have been reported for model cyanobacterial species (e.g., Xu et al., 2003; Cohen et al., 2004; Dashdorj et al., 2005; Milanovsky et al., 2014; Sun et al., 2014; Makita and Hastings, 2015), although more even utilization has been also reported for these organism too (e.g., Santabarbara et al., 2006, 2010c). For the modeling described here we consider even initial populations on the phylloquinones ($\left[{\text{A}}_{1\text{A}}^{-}\right]\left(0\right)=\left[{\text{A}}_{1\text{B}}^{-}\right]\left(0\right)=$0.5) and no population in all other intermediates. This scenario is more representative, in general, to the one most commonly encountered in green algae. It was chosen because some of the site-directed mutants discussed below were raised only in the PSI of *C. reinhardtii*. It is worth mentioning, however, that changes in the initial populations would only affect the relative amplitudes of ${\text{A}}_{1}^{-}$ oxidation phases, but not their lifetimes.

## Kinetic Description of A_1_ Oxidation

Kinetic modeling of electron transfer reactions, coupled to theory-based ET rate description, represents an useful tool to extract the energetics associated to reactions whose chemical-physical properties are cumbersome to address by other biophysical and biochemical methodologies. Yet, as discussed in the previous paragraphs, the description of the ET rates also requires the knowledge of a set of parameters (e.g., $\Delta G_{DA}^{0}$, λ_*DA, tot*_, ${\left|H_{DA}\right|}^{2}$, and $\hbar {\bar{\omega }}_{DA}$). These are not always straightforwardly extractable from the experimental data and need therefore to be fixed in the calculation, often to some consensus values stemming from the parameterisation developed by Moser and Dutton (1992) and Moser and Dutton (1996). Because of these limitations, it appears sensible to limit the tuning of adjustable parameters to reach a good semi-quantitative description of the results rather than aiming at an exact reproduction of the experimental data.

In the following paragraphs the discussion will be focused on the kinetics of $A_{1}^{-}$ oxidation in PSI, and in particular on the driving force (standard free energy differences, $\Delta G_{{\text{A}}_{\text{1}}}^{0}$) associated to these reactions, while limiting to a minimum the tuning of all other adjustable parameters, particularly the reorganization energies. Thus, the description presented shall be considered to only semi-quantitatively describe these ET reaction kinetics, and whenever possible the values of parameters, unless taken from independent studies, will be rounded to the closer decade interval. It is arguable that this is a reasonable approach, not only because of the uncertainties on some of the parameters already addressed but also because, whereas the modeling provides information on the population evolutions (changes in cofactor redox-state concentration over time), the experimentally measured kinetics also depend on other properties of the cofactors. For instance, in transient absorption, which is possibly the most popular experimental approach, the experimental data also reflects the differential (reduced/oxidized) extinction coefficient of the cofactors (Δε). This is expected to be similar, but not necessarily identical, for the case of the phylloquinones considered here. Moreover, contributions of other redox centers, such as the iron-sulfur centers, and the occurrence of spectral changes associated with electrogenic reactions (electrochromism), would also give rise to kinetically overlapped optical transients, so that an exact correspondence between the population evolution and the experimental shall not be, in principle, expected.

Moreover, recent reinvestigations have shown that ET oxidation kinetics display significant dispersive character even at room temperature (Malferrari et al., 2016; Kurashov et al., 2018), i.e., are better described by lifetime distributions rather than a sum of discrete exponentials. This implies some distributed character also for the microscopic parameters employed in the ET rate description. Instead, in the modeling approach described in Paragraph 3, the parameters are accounted by a single, well-defined, value instead. Hence, the retrieved values for the parameter of interest ($\Delta G_{{\text{A}}_{\text{1}}}^{0}$, in this case) shall better be considered as an estimation of its mean value, provided that the underlying distribution is symmetric and reasonably narrow.

Thus, within this semi-quantitative limit, two alterative energetic scenarios for $A_{1}^{-}$ oxidation in PSI will be discussed: (i) the one involving a “weak driving force” for, particularly, $A_{1\text{A}}^{-}$ oxidation (ii) the one considering “large driving force” for the oxidation of both $A_{1\text{A}}^{-}$ and $A_{1\text{B}}^{-}$.

### Wild-Type Reaction Centers

#### Weak Driving Force Case

**Figure 3:**
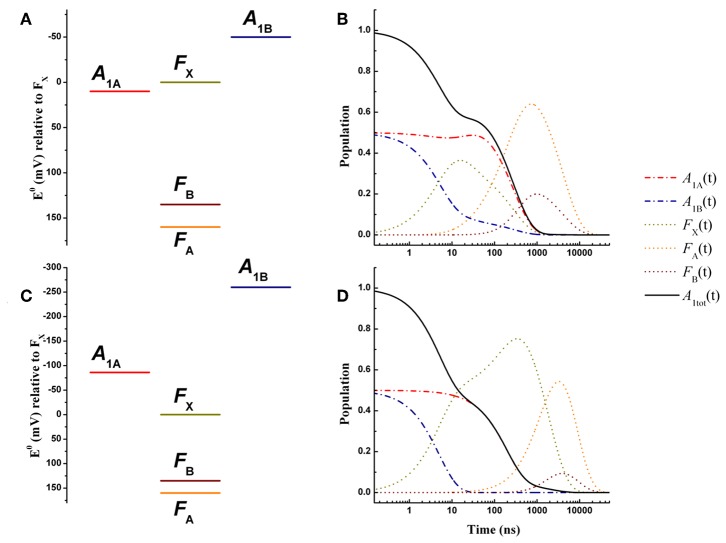
Energetics **(A,C)** and kinetic simulations **(B,D)** of the electron transfer reactions downstream of *A*_1_ for a wild-type PSI reaction center, according to the “weak” **(A,B)** and the “large” **(C,D)** driving force scenarios. Dash-dotted blue lines: *A*_1B_; dash-dotted red lines: *A*_1A_; dotted golden lines: *F*_X_; dotted orange lines: *F*_A_; dotted burgundy lines: *F*_B_. Black solid line: *A*_1tot_ = *A*_1A_+*A*_1B_.

The energetic scenario describing the oxidation kinetics of $A_{1\text{A}}^{-}$ and $A_{1\text{B}}^{-}$ in a wild-type PSI reaction center, within the weak driving force framework, is schematically illustrated in Figure 3A. It is considered that $\Delta {\text{G}}_{{\text{A}}_{1\text{A}}}^{0}$ = 10 meV, $\Delta {\text{G}}_{{\text{A}}_{1\text{B}}}^{0}$ = −50 meV, $\Delta {\text{G}}_{{\text{F}}_{\text{X}}}^{0}$ = −160 meV, and $\Delta {\text{G}}_{{\text{F}}_{\text{A}}}^{0}$ = 25 meV, values that fall within the range originally suggested, within the bidirectional ET framework, by Santabarbara et al. (2005a) and which validity has been recently rediscussed (Santabarbara et al., 2019). However, similar scenarios for $A_{1\text{A}}^{-}$ oxidation were previously suggested by Brettel (1997). Setting the redox midpoint potential of *F*_B_ to −555 mV, which is compatible with the direct redox titration (*see* Brettel, 1997 for a compilation of literature values), the redox potentials of the other considered cofactors are: $E_{{\text{A}}_{1\text{A}}}^{0}$ = −680 meV, $E_{{\text{A}}_{1\text{B}}}^{0}$ = −740 mV, $E_{{\text{F}}_{\text{X}}}^{0}$ = −690 mV, and $E_{{\text{F}}_{\text{A}}}^{0}$ = −530 mV. The redox midpoint potential of $P_{700}^{+}$ is taken as 450 mV. In order to reduce to a minimum the number of adjustable parameters, a common value of the total reoganization energy (λ) equal to 0.7 eV was employed for all forward reactions involving $A_{1\text{A}}^{-}$, $A_{1\text{B}}^{-}$, and *F*_X_. A larger reorganization energy (0.825 eV) was utilized for ET reactions between the remaining metal centers (*F*_A_ and *F*_B_) instead and for *all* the recombination reactions with $P_{700}^{+}$. This reorganization energy might appear low, as Density Function Theory (DFT) calculations performed on Ferredoxin, which is highly homologous to the *F*_A_/*F*_B_-binding PsaC subunit of PSI, predicted it to be as high as 0.64 for the internal reorganization component alone (Sigfridsson et al., 2001). Hence, calculations were also performed by employing total reorganizations of 1 and 1.3 eV (~twice the internal) for the ET between *F*_A_ and *F*_B_. These simulations, shown in Figure S1, indicate that the $A_{1}^{-}$ oxidation kinetics remain virtually unchanged, but significant kinetic slowdown is modeled for the downstream cofactors kinetics. Whereas, a value of λ_*F*_*A*_→*F*_*B*__ = 1 eV still provides acceptable descriptions, raising its value to 1.3 eV resulted in a too slow oxidation of the terminal iron-sulfur cluster acceptors (without changing any of the other parameters). The only rate constant which was not modeled according to theory was *F*_B_ oxidation, as this represents just an output from the system. The value of this rate was set to (1 μs)^−1^ corresponding to the fastest phase of Ferredoxin reduction (Setif, 2001). The list of all parameters used in the simulations is reported in Table 1.

The simulated ET kinetics of all the intermediates considered employing the just described parameters are shown in Figure 3B. The *A*_1A_ and *A*_1B_ oxidation kinetics are characterized by four main exponential decay components described by lifetimes of 5.2, 23.1, 131, and 285 ns (Table 2), whereas an additional lifetime component of ~4 μs has extremely low amplitude (note that the lifetimes are common to *all* intermediates, but not the amplitudes). The relative amplitudes of these components, for the total oxidation of $A_{1}^{-}\left(t\right)$, which is the only experimental accessible one and which is given by the straight summation of modeled $A_{1\text{A}}^{-}\left(t\right)$ and $A_{1\text{B}}^{-}\left(t\right)$, are 0.45, −0.12, 0.0007, and 0.66 (Table 2). This indicates that the fastest and slowest components are largely dominant. As discussed in previous studies (Santabarbara et al., 2010a, 2015; Santabarbara and Zucchelli, 2016), the number of simulated components is larger than those retrieved experimentally. This is because the simulations will yield a number of lifetimes equal to the number of ET steps considered. Still, predicted lifetimes which fall closely spaced in time (e.g., ~5 and 25 ns) would be hardly distinguishable experimentally. Similar considerations could be extended to the ~130 and 280 ns component, due also to the differences in relative amplitudes. Nonetheless an $A_{1}^{-}$ oxidation phase characterized by a lifetime of 140–180 ns became observable in mutants in which the ~250 ns phase was lengthened as a result of specific amino-acid side chain substitutions (Byrdin et al., 2006; Santabarbara et al., 2008, 2015). Moreover, this phase was also observed in temperature dependence studies of $A_{1}^{-}$ oxidation of wild-type PSI (Agalarov and Brettel, 2003; Santabarbara et al., 2009), suggesting it was not the result of mutations of the PhQ binding site. The simulated lifetime of 131 ns corresponds therefore rather nicely to the experimentally retrieved values. Noteworthy, whereas the ~5, 25, and 250 ns are simulated also in minimal models considering only *A*_1A_ and *A*_1B_ and *F*_X_ (e.g., Santabarbara et al., 2010a, 2015; Santabarbara and Zucchelli, 2016), the 131 ns is not, indicating it is associated to ET reactions involving the terminal 4Fe-4S cluster acceptors. In fact, although this component has rather weak amplitude on $A_{1}^{-}\left(t\right)$ it is a dominant one in oxidation of *F*_B_. Moreover, it is the lifetime associated to this component, together with its relative amplitude in the Fe-S clusters oxidation, which is primarily affected by varying the value of λ_*F*_*A*_→*F*_*B*__, becoming ~500 ns and ~1 μs for total reorganizations of 1 and 1.3 eV, respectively (Figure S1). Grouping the lifetimes of <50 ns and more than 50 ns into macroscopic “fast” and “slow” oxidation phases, respectively, a proportion of 0.34:0.66 between them is found, corresponding to an average lifetime of 188 ns, which is in good agreement with the experimental reports, particularly concerning PSI of green algae (Santabarbara et al., 2005a, 2010a; Rappaport et al., 2006).

**Table 2:** Principal simulation outputs describing $A_{1}^{-}$ oxidation kinetics in wild-type PSI.

	**Weak driving force**			**Large driving force**			
****τ** (ns)**	***p*_*A*_1A__**	***p*_*A*_1B__**	***p*_*A*_1tot__**	****τ** (ns)**	***p*_*A*_1A__**	***p*_*A*_1B__**	***p*_*A*_1tot__**
5.21	0.073	0.384	0.458	5.13	0.00052	0.500	0.500
23.2	−0.1644	0.0458	−0.119	188	0.455	−0.00016	0.455
131	0.0006	0.0001	0.0007	499	2.0E-06	9.6 10^−09^	2.1 10^−06^
285	0.588	0.0694	0.657	1,873	0.0438	0.0003	0.0441
4017	0.0029	0.0004	0.0033	6,211	1.7 10^−04^	1.2 10^−06^	1.7 10^−04^

*Summary of the simulated lifetimes (τ) and amplitudes (p) associated to each component describing the population evolution of the phyllosemiquinones $A_{1\text{A}}^{-}\left(t\right)$ and$A_{1\text{B}}^{-}\left(t\right)$, as well as the total population evolution given by $A_{1,tot}^{-}\left(t\right)$ = $A_{1\text{A}}^{-}\left(t\right)$+$A_{1\text{B}}^{-}\left(t\right)$*.

Recombination reactions (from all considered co-factors) were modeled simply suppressing the output from the system. Under these conditions the only possible oxidation path for electrons that have reached the terminal acceptors is that of recombining with the cation located on *P*_700_. It is obtained that under these simulation conditions, *F*_A_ and *F*_B_ are oxidized with mean lifetimes of ~75 ms, equalling $P_{700}^{+}$ reduction. This value is also in excellent agreement with experimental estimations, from which a rather broad range of 20–100 ms is reported (Brettel, 1997; Shinkarev et al., 2002). Importantly, the recombination lifetime is much faster than the predicted rates for direct recombination between either *F*_A_ and *F*_B_ and *P*_700_ (which are in the hours-to-days span due to large distances between the redox centers). Thus, the modeled recombination has to proceed by back-population (reduction) of the co-factors upstream to terminal iron-sulfur clusters, and is therefore kinetically limited by the large energy gap between *F*_A_ and *F*_X_.

#### Large Driving Force Case

An energetic scheme and the associated kinetic simulations representative of the large driving force scenario for the oxidation of both of $A_{1\text{A}}^{-}$ and $A_{1\text{B}}^{-}$ in a wild-type PSI are presented in Figures 3C,D, respectively. The values of $\Delta {\text{G}}_{{\text{A}}_{1\text{A}}}^{0}$ = −86 meV, $\Delta {\text{G}}_{{\text{A}}_{1\text{B}}}^{0}$ = −259 meV were taken from the study of Ptushenko et al. (2008). To allow a straightforward comparison, all other parameters, with the exception of λ, were the same as in the simulations of Figure 3B. In order to semi-quantitatively describe $A_{1}^{-}$ oxidation, a value of λ = 1 eV (rather than 0.7 eV used for the weak driving force scenario) needed to be considered, and to limit the number of tuneable parameters this value was adopted for all others reaction (forward and recombination) considered (Table 1). Simulations for the values proposed by Milanovsky et al. (2017), $\Delta {\text{G}}_{{\text{A}}_{1\text{A}}}^{0}$ = −55 meV, $\Delta {\text{G}}_{{\text{A}}_{1\text{B}}}^{0}$ = −220 meV, in which the driving forces are lower but remain significant, are presented in Figure S2, and with all other relevant parameters listed in Table S1. Semi-quantitative agreement was reached just by using a slightly lower value of the total reorganization energy (0.925 eV), common to all considered reactions.

The kinetics are described by three sub-microsecond components of 5.1, 194, and 499 ns, and two microsecond phases of 1.9 and 6 μs, with the longest one representing the outcome from the system (Table 2). The two fastest components have by far the largest amplitude on the total $A_{1}^{-}$ oxidation, corresponding to 0.5 and 0.45, respectively, with the residual 0.05 being associated to the 1.9 μs phase, which is not reported in experimental measurements, but it may simply escape detection because of its very small amplitude (Table 2). The 499 ns phase corresponds to inter Fe-S ET. Its lifetime matches the one also predicted for the weak-driving force scenario when using the same value of 1 eV for λ_*F*_*A*_→*F*_*B*__, and it can be accelerated to resemble the experimental reported phase of ~160 ns by decreasing the reorganization to ~0.85 (not shown). However, this would assume that λ_*F*_*A*_→*F*_*B*__ has a lower value than λ_*A*_1_→*F*_*X*__, which is not reasonable as ET between metal cluster is expected to be associated with larger reorganization energies. Differently from the weak driving force scenario, the relative amplitudes of the fast and slow $A_{1}^{-}$ oxidation phases, match the initial populations.

Hence, both the weak driving force and the large driving force scenarios appear to provide an overall consistent description of the ET kinetics of forward reactions in wild-type PSI reaction center. Within this simple framework, however, the weak driving force model reproduce more satisfactorily the recombination reactions from the terminal Fe-S cluster [see discussion in the Supplementary information of Santabarbara et al. (2019)]. Thus, this energetic scheme can be considered as a good starting point to address the effect of specific mutations at the level of the phylloquinone binding site. A comparison with the shifts in driving force necessary to account for the kinetic alterations observed in the mutants within the large driving force scenario, will however be discussed.

### Effect of Mutations of the Phylloquinone Binding Pockets

The energetics of $A_{1}^{-}$ oxidation has been altered by two complementary approaches, one involving the perturbation of PhQ-protein interactions by mutations of key residues in the redox center binding niches (Santabarbara et al., 2005a, 2010a; Rappaport et al., 2006; Srinivasan and Golbeck, 2009), the other involving the replacement of the naturally occurring PhQ with exogenous quinones, possessing inherently different redox potentials (Iwaki et al., 1996; Itoh et al., 2001; Makita et al., 2015; Milanovsky et al., 2017). One of the principal differences between the two strategies is that whereas in the “mutational” approach the properties of *A*_1A_ and *A*_1B_ can be tested separately by specific mutations of either the PsaA or the PsaB subunit (moreover mutants of *both* subunits have also been reported (Guergova-Kuras et al., 2001; Rappaport et al., 2006; Santabarbara et al., 2010a), in the “quinone replacement” approach, the energertics of both ET branches are simultaneously affected, as it is the redox properties of the exogenous moiety which governs the perturbation. Results from the quinone-exchange approach have been recently reviewed in detail by Makita and Hastings (2016), Makita and Hastings (2017), and Milanovsky et al. (2017). Therefore, here we focus the discussion on the effect of site directed mutants of the *A*_1_ binding pocket.

Site-directed mutations altering the PhQ binding niches of both the PsaA and PsaB subunits have been investigated in a rather large number of studies, using as model organisms mainly *Synechocystis sp*. PCC6803 for cyanobacteria and *Chlamydomonas reinhardtii* for green algae (see Santabarbara et al., 2005a, 2010a; Rappaport et al., 2006; Redding and van der Est, 2006; Srinivasan and Golbeck, 2009 for reviews). In general, mutants of the PsaA subunit, and therefore perturbations of *A*_1A_, appear to be better characterized, because of the slower apparent “natural” oxidation kinetics at room temperature and also because recombination reactions with $P_{700}^{+}$ at cryogenic temperatures (Brettel, 1997; Shinkarev et al., 2002), at least under ambient redox conditions (Poluektov et al., 2005; Santabarbara et al., 2005b, 2006, 2010c), are dominated by the $A_{1\text{A}}^{-}$. Henceforth, the discussion and the accompanying numerical simulations will be focused on mutations of the PsaA subunit, although similar arguments could be applied to the mutants of PsaB affecting principally *A*_1B_. The effect of specific amino-acid substitutions in the *A*_1A_ binding niche can be classified in three main “kinetic phenotypes”.

Most mutants were designed to target residues which appear to closely interact with the PhQ moiety, according to structural models (Figure 1B). The main interactions are predicted to be due to aromatic π-stackings (such as Trp697) and, more indirectly, from the H-bond network amongst residues composing the binding niche [e.g., Met688, Ser692, Trp697, numbering as in *Thermosynechococcus elongatus* structural model of Jordan et al. (2001)]. Substitutions of these residue side chains led to a slowing down of the 200–300 ns oxidation lifetime observed in the wild-type to 600–1,200 ns in mutants of both green algae (e.g., Guergova-Kuras et al., 2001; Muhiuddin et al., 2001; Purton et al., 2001; Ali et al., 2006; Byrdin et al., 2006; Santabarbara et al., 2008) and cyanobacteria (e.g., Xu et al., 2003). Yet, the relative amplitudes of “fast” and “slow” phases of $A_{1}^{-}$ oxidation were hardly affected by these mutations (Guergova-Kuras et al., 2001; Byrdin et al., 2006; Rappaport et al., 2006; Santabarbara et al., 2008, 2010a). This group of mutants, which comprises a large number of specific residue substitutions, will be considered, also in the accompanying calculations, as a single class and referred to as “moderately slowing down” scenario.

An extreme slowing down effect was observed in a specific mutant of the PsaA subunit of *C. reinhardtii*, PsaA-F689N (Santabarbara et al., 2015). This residue is likely involved in “distal” π-stacking and its substitution with an amino-acid bearing a polar side chain resulted in an apparent $A_{1\text{A}}^{-}$ oxidation lifetime of ~17 μs, i.e., 20 times slower than the wild-type. This rather unique, at least so far, mutation effect will be considered as an independent class, referred hereafter as “extreme slow-down” scenario.

The last class of mutants which will be considered comprises substitutions of residues putatively involved in the asymmetric H-bond to the PhQ moiety. According to the structural models, H-bonding will take place through Leu722 peptide bond (Santabarbara et al., 2010b; Srinivasan et al., 2011; Mula et al., 2012). Henceforth, side-chains substitutions, excluding the one with Proline, are not expected to *directly* perturb the protein-cofactor interactions. Yet, because of the tight-packing of the side chains, mutations of Psa-L722 might indirectly perturb the H-bond donation to *A*_1A_ as a result of steric hindrance and consequent micro-reconfigurations of the binding-pocket. Indeed, mutations of the Leu722 residue of the PsaA subunit in both cyanobacteria (Srinivasan et al., 2011) and green algae (Santabarbara et al., 2010b) led to alterations of the $A_{1\text{A}}^{-}$ oxidation kinetics that, differently from all mutations discussed above, decreased the oxidation lifetime to 160–180 ns. Moreover, a redistribution of the “fast”:“slow” phase amplitudes was detected resulting in a relative increase of the rapid phase and therefore in a significant acceleration of the overall oxidation kinetics (Santabarbara et al., 2010b; Srinivasan et al., 2011). This third class of mutants will be hereafter referred to as “moderately speed up” scenario.

**Figure 4:**
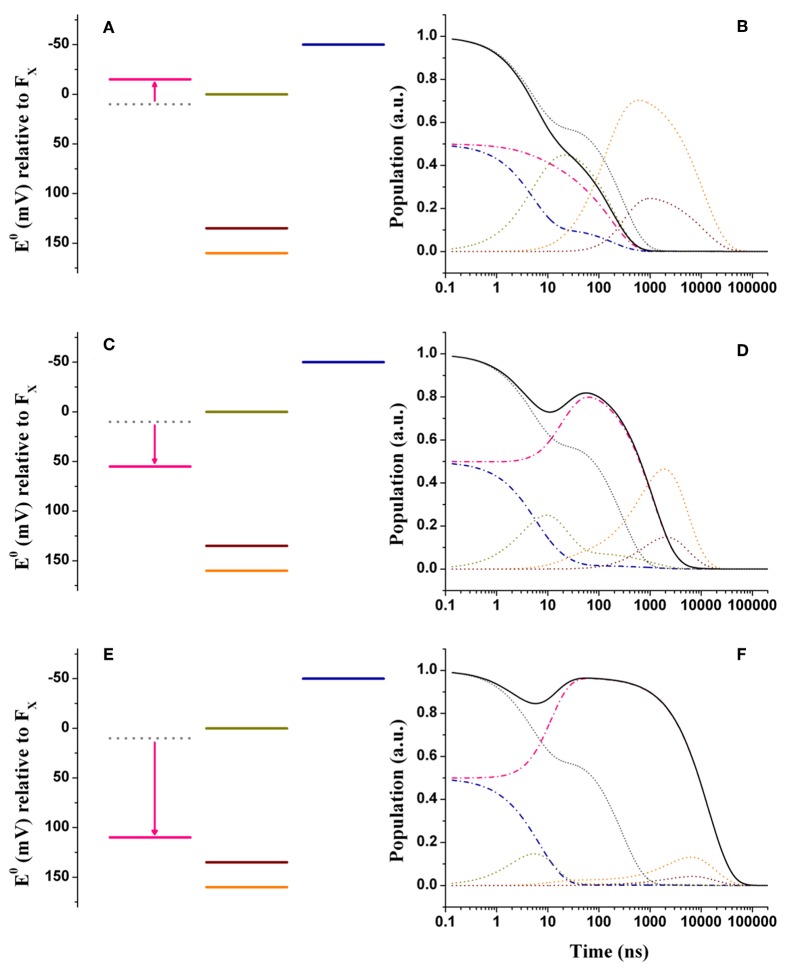
Energetics **(A,C,E)** and kinetic simulations **(B,D,F)** of the electron transfer reactions downstream of *A*_1_ in the three classes of mutants of the PsaA reaction center subunit discussed in the text. **(A,B)** Simulate the “moderately speed up” scenario; **(C,D)** The “moderately slowing down” scenario; **(E,F)** The “extreme slow-down” scenario. In all energetics panels the shifted redox midpoint potential induced by the specific mutation is indicated in pink, whereas the wild-type potential is shown as gray dotted lines. In the kinetic simulations the same color-coding as in Figure 3 is used (dash-dotted blue lines: *A*_1B_; dotted golden lines: *F*_X_; dotted orange lines: *F*_A_; dotted burgundy lines: *F*_B_; black solid line: *A*_1tot_ = *A*_1A_+*A*_1B_) for the exception of the “mutated” *A*_1A_ kinetics which are shown in pink. The gray dotted lines show the simulations of *A*_1tot_ relaxation in the wild-type for ease of comparison.

In order to rationalize the kinetic perturbations in the three classes of mutants discussed above, simulations were performed starting from the wild-type scenarios, presented in Figure 3. It is here considered that the main impact of the mutations is on the redox midpoint potential of $A_{1\text{A}}^{-}$, thereby determining a variation of the Gibbs free energy $\Delta G_{A_{1\text{A}}}^{0}$. Thus, we introduce the term $\Delta \Delta G_{A_{1\text{A}}}^{0}$ to specifically highlight the effect of the amino-acid substitutions. Figures 4A,B show the alterations to the system energetics necessary to simulate the kinetics for the PsaA-L722 mutants belonging to the moderately accelerating mutant class when starting from the weak driving force scenario (Figure 3A). By considering $\Delta \Delta G_{A_{1\text{A}}}^{0}$ = −25 meV, i.e., a net increase in the driving force, the acceleration of $A_{1\text{A}}^{-}$ oxidation kinetics with respect to the WT is reproduced rather well, as the simulated lifetimes become 5.2, 22.5, 141, and 182 ns. Interestingly, also the redistribution in amplitudes between the “fast” and the “slow” phases of $A_{1}^{-}$ oxidation is semi-quantitatively reproduced in the simulation. The shift in $\Delta G_{A_{1\text{A}}}^{0}$reported here is similar to that already discussed by Santabarbara et al. (2010b) with small differences related to adopting an extended kinetic model as well as specific values of $\hbar \bar{\omega }$ in the present calculations. Thus, whereas the acceleration of the kinetics can be straightforwardly linked to the increase in the driving force, the apparent amplitude redistribution results from the suppression of the transient inter-quinone electron transfer driven by the asymmetry in $\Delta G_{A_{1\text{A}}}^{0}$ and $\Delta G_{A_{1\text{B}}}^{0}$ that is present in the wild-type and that is reduced in the mutants, as the redox gap between the two quinones becomes less significant (Santabarbara et al., 2010b). The same shift in the reaction free energy,$\Delta \Delta G_{A_{1\text{A}}}^{0}$ = −25 meV, satisfactorily describes the faster lifetime in the PsaA-L722 also within the large driving force framework. However, in this case, it does not account for the just discussed amplitude redistribution. The simulations for the mutants starting from the wild-type scenario discussed by Ptushenko et al. (2008) and Milanovsky et al. (2017) are presented in Figures S3A,B, S4A,B, respectively.

Figures 4C,D show the simulations for the class of “moderately slowing down” mutants, targeted to obtain a main oxidation lifetime for $A_{1\text{A}}^{-}$ of about 1,000 ns. The effect of the mutations can be rather well-simulated by considering $\Delta \Delta G_{A_{1\text{A}}}^{0}$ = +45 meV. For this alteration of $\Delta G_{A_{1\text{A}}}^{0}$, lifetimes of 4.9, 16, 131, and 968 ns were simulated, which correspond rather well with experimental observations especially considering that those should be taken as representative of several different specific mutations. One interesting observation is that the ~131 ns component predicted in the wild-type is hardly affected by the shift in $\Delta G_{A_{1\text{A}}}^{0}$. However, whereas this component falls quite close to the 150 ns oxidation phase in the wild-type, it becomes temporally well-separated in the mutants. This agrees with the observation that an intermediate phase of similar lifetime (~150 ns) could be also retrieved in mutants that slowed down $A_{1\text{A}}^{-}$ oxidation (Byrdin et al., 2006; Santabarbara et al., 2008). The simulations predict however a redistribution (~15%) of the amplitudes in favor of the slower decay phases, which somewhat exceeds the virtual absence of redistribution reported for experimental data. From a semi-quantitative point of view, these simulations can nonetheless be considered satisfactory.

The effect of mutants that moderately slow down the $A_{1\text{A}}^{-}$ oxidation phase is more difficult to reproduce in the large driving force scenario. Lowering the driving force leads to both a lengthening of the ~200 ns phase observed in the wild-type as well as a sizable increase of the ~2 μs contribution, which is instead negligible in the unperturbed system. In this case we opted then to match the lengthening in the average oxidation lifetime which is accounted by considering +35 < $\Delta \Delta G_{A_{1\text{A}}}^{0}$ <55 meV (Figures S3C,D, S4C,D) similarly to what derived from the weak driving force model.

In Figures 4E,F are shown the energetic scenario and the simulated kinetics describing the “extreme slowing” of $A_{1\text{A}}^{-}$ oxidation reported for the PsaA-F689N mutant. A remarkable $\Delta \Delta G_{A_{1\text{A}}}^{0}$ = +120 meV is required to describe this class of mutants, yielding lifetimes of 3.8, 9.2, 131 ns, and 3.6, 11.2 μs. In this scenario, the slowest (11.2 μs) component carries significant amplitude and needs therefore to be considered. In fact, its simulated value corresponds rather well with the one observed experimentally. As already discussed in Santabarbara et al. (2015) however, when considering only a shift in $\Delta G_{A_{1\text{A}}}^{0}$, an excessive redistribution of the relative amplitudes with respect to those observed experimentally is simulated. This discrepancy can be compensated when taking into account possible effects of the mutation also on the reorganization energy, because of the very polarisable nature of this particular substitution. Since an exact description goes beyond the scope of the present survey, it shall be argued that a decrease of about 100 meV in the $A_{1\text{A}}^{-}$ oxidation driving force shall be considered as an upper limit for the mutation-induced perturbation.

When considering shifts in the driving force in the +110 < $\Delta \Delta G_{A_{1\text{A}}}^{0}$ <130 meV interval, a good description of the PsaA-F689N mutation effect is obtained also within the large driving force wild-type scenarios (Figures S3E,F, S4E,F). Also starting from these initial models a large redistribution of amplitudes is simulated, since, in the mutant, $A_{1\text{A}}^{-}$ oxidation becomes energetically unfavorable, thereby promoting inter-quinone initial population transfer.

## General Perspectives

From the above reported simulations it can be concluded that, when considering initial energetic scenarios in which $A_{1}^{-}$ oxidation is associated with either weak or rather large driving forces, the effect of mutations of key residues participating to the $A_{1\text{A}}^{}$ binding site can be reasonably rationalized assuming that alterations of the phylloquinone redox properties represent the dominant factor. Substitutions of conserved residues in the $A_{1\text{A}}^{}$ binding niche accounted for a shift of about ~+40 mV of the phylloquinone potential, as long these involved conservative side chain exchanges that did not include additional highly polar or possibly charge-bearing side chains. The latter can lead instead to much larger perturbations, as observed in the PsaA-F689N mutant of *C. reinhardtii*. Mutations of the residues involved in H-bond donation to PhQ can lead to similar, absolute value, potential shift but toward a higher reducing potential ($\Delta \Delta G_{A_{1\text{A}}}^{0}$ = −25 meV). This can result from a combination of eventually antagonistic effects due to changes in both the H-bond strength (likely a weakening) as well as to more indirect perturbations of the binding site. Interestingly, whereas these mutants showed faster oxidation kinetics, which could be seen as an improvement of the overall reaction center performance, they displayed, at the same time, a lowering of the PhQ binding strength, manifesting as a RC sub-population with empty binding sites. Recombination reactions leading to triplet state formation were detected in this fraction of center (Santabarbara et al., 2010b; Srinivasan et al., 2011). Since this can lead to oxidative stress because of singlet oxygen sensitization, a tighter PhQ binding appears as a weak penalty in place of an overall marginal kinetic slowing.

Still, although a good semi-quantitative description of both the ET kinetics in the wild-type as well as in mutations affecting PhQ binding interactions is obtained, further elucidation concerning the *precise* energetics of *A*_1_ oxidation as well as of the successive ET reactions, particularly those involving the iron-sulfur clusters which are instead significantly different depending on the initial energetic scenarios adopted for the wild-type description, is still need. It can be foreseen that this shall be accomplished by a combination of experimental approaches, ranging from quantum-chemical methods to spectroscopic ones. Concerning the latter, considering that a wide range of mutations has been already engineered, and that quinone moiety substitutions represent a power tool to alter the PhQ energetics as well, a systemic joint experimental and kinetic modeling analysis, not only of forward electron transfer reactions at room temperature, but also of their temperature dependence, that has been very seldom explored, and of the recombination reactions with $P_{700}^{+}$, together with their temperature dependence, would not only almost certainly result in a far more precise description of the system energetics but also in a better understanding of the protein-mediated tuning of ET co-factors.

## Author Contributions

SS and AC wrote the paper. SS performed the kinetic simulations.

### Conflict of Interest Statement

The authors declare that the research was conducted in the absence of any commercial or financial relationships that could be construed as a potential conflict of interest.
