# Supplementary material for: Kinetics and Energetics of Phylloquinone Reduction in Photosystem I: Insight From Modeling of the Site Directed Mutants
Source: Front Plant Sci. 2019 Jul 2;10:852. doi: 10.3389/fpls.2019.00852 (PMC6614487; doi:10.3389/fpls.2019.00852)
Supplement: Supplementary file 1 [file Data_Sheet_1.docx]

Supplementary Material

**S1. MODELLING OF $A_{1}^{-}$ OXIDATION WITHIN THE WEAK DRIVING FORCE ENERGETIC SCENARIO: IMPACT OF THE REORGANISATION ENERGY ASSOCIATED TO $F_{A}\to F_{B}$ ELECTRON TRANSFER.**

Figure S1 shows the simulated population evolutions of reduced ET cofactors $A_{1\text{B}}^{-}$ (dash-dotted blue lines), $A_{1\text{A}}^{-}$ (dash-dotted red lines), *F*_X_ (dotted golden lines), *F*_A_ (dotted orange lines) and *F*_B_ (dotted burgundy lines) according to the energetic model presented in Figure 3A and Table 1 of the main body, but increasing the value of the reorganisation energy ($\lambda$) for the electron transfer between the terminal electron acceptors *F*_A_ and *F*_B_ from 0.825, as shown in Figure 3B, to 1 eV (Figure S1A) and 1.3 eV (Figure S1B).

To allow for direct comparison, the modelled total population evolution of phyllosemiquinones, $A_{1,tot}^{-}$=$A_{1\text{A}}^{-}$+$A_{1\text{B}}^{-}$ (black solid line), is compared to the one obtained for $\lambda_{F_{A}\to F_{B}}$=0.825 eV (open grey circles). It is then clear that increasing the value of $\lambda_{F_{A}\to F_{B}}$ has virtually no effect on $A_{1,tot}^{-}$ population evolutions.

However, it has a significant impact on downstream electron transfer. The slowing down of reduced *F*_A_ oxidation (dotted orange lines) brought about by increasing $\lambda_{F_{A}\to F_{B}}$ from 1 eV to 1.3 eV is immediately obvious by inspecting panel A and B of Figure S1. To allow better comparison with the simulations presented in the main body (Figure 3B), the total population evolution of the Fe-S ($FeS_{tot}$=*F*_X_+*F*_A_+*F*_B_) is also shown in Figure S1. The solid light-orange line corresponds to *FeS*_tot_ in the simulation for $\lambda_{F_{A}\to F_{B}}$=1 eV (S1A) and $\lambda_{F_{A}\to F_{B}}$=1.3 eV (S1B), whereas the results obtained for $\lambda_{F_{A}\to F_{B}}$=0.825 eV are also plotted (dashed line and open circles, light orange). Considering $\lambda_{F_{A}\to F_{B}}$=1 eV (S1A) still provides a reasonable description despite slightly slowing the total FeS oxidation. Yet, a further increase to 1.3 eV still provide an acceptable FeS reduction description, but their overall oxidation is not compatible with the experimental observables.

**S2. MODELLING OF $A_{1}^{-}$ OXIDATION WITHIN THE LARGE DRIVING FORCE ENERGETIC SCENARIO (MILANOVSKY ET AL. 2017).**

Figure S2 shows the energetic scheme and the modelled population evolutions, resulting from considering the free energies for phyllosemiquinone oxidation $\Delta G_{1\text{A}}^{0}$= –50 meV and $\Delta G_{1\text{B}}^{0}$= –220 meV suggested by Milanovsky and coworkers (2017). This still represent a large driving force scenario, but with lower driving force with respect to those proposed by Ptushenko et al. (2008) already presented in the main body of this article (Figures 3C and D). The remaining free energy differences ($\Delta G_{FX}^{0}$, $\Delta G_{FA}^{0}$) were the same as in Figure 3. A common value of the reorganisation energy $\lambda$=0.925 eV was used for the forward and recombination reactions, thereby reducing the number of adjustable simulation parameters. The remaining parameters, common to all reactions were the electronic coupling matrix element, $\left|H_{0}\right|^{2}$=1.3 10^-3^ eV^2^, the barrier damping factor,$\beta$=1.34 Å^-1^, and the temperature, *T*=290 K. Rates were calculated according to Equation 1 in the main text.

Figure S2. Energetics (**A**) and kinetic simulation (**B**) of the electron transfer reactions downstream of *A*_1_ for the large driving force scenario of Milanovsky et al. (2017). Dash-dotted blue lines: *A*_1B_; dash-dotted red lines: *A*_1A_; dotted golden lines: *F*_X_; dotted orange lines: *F*_A_; dotted burgundy lines: *F*_B_. Black solid line: $A_{1,tot}^{-}$=$A_{1\text{A}}^{-}$+$A_{1\text{B}}^{-}$.

**Table S1. Principal simulations output for ET in WT PSI.**

Lifetimes (τ) and amplitudes (*p*) associated to each component describing the population evolution of the phyllo(semi)quinones (i.e. $A_{1\text{A}}(t)$ and $A_{1\text{B}}(t)$) as well as the total population evolution given by $A_{1tot}(t)=A_{1\text{A}}(t)+A_{1\text{B}}(t)$.

**S3. MODELLING THE EFFECTS OF MUTATIONS IN THE *A*_1_ BINDING NICHE WITHIN THE LARGE DRIVING FORCE ENERGETIC FRAMEWORK.**

Figures S3 and Figure S4 show the simulations of the kinetic effects brought about by single-residue substitutions in phylloquinone binding niche of the PsaA subunit, leading to moderate speeding up (PsaA-L722), moderate slowing down (e.g. PsaA- Met688/Ser692/Trp697,) and extreme slowing down (PsaA-F689N) of the $A_{1}^{-}$ oxidation, starting from the large driving force wild-type scenario proposed by Ptushenko et al. (2008), Figure S3, and Milanovsky et al. (2017), Figure S4.

**Figure S3.** Energetics (A, C, E) and kinetic simulations (B, D, F) of the electron transfer reactions downstream of *A*_1_ in the three classes of mutants of the PsaA reaction centre subunit, starting from the free energy = – 86 meV and = –259 meV in the wild-type (Ptushenko et al. 2008). **A** and **B** simulate the “moderately speed up” scenario, ($\Delta\Delta G_{A_{1\text{A}}}^{0}$= – 25 meV); **C** and **D** the “moderately slowing down” scenario ($\Delta\Delta G_{A_{1\text{A}}}^{0}$= + 55 meV); **E** and **F** the “extreme slow-down” scenario ($\Delta\Delta G_{A_{1\text{A}}}^{0}$= + 150 meV). In all energetics panels the shifted redox midpoint potential induced by the specific mutation is indicated in pink, whereas the wild-type potential is shown as grey dotted lines. In the kinetic simulations the same colour-coding as in Figure 3, S1 and S2 is used (dash-dotted blue lines: *A*_1B_; dotted golden lines: *F*_X_; dotted orange lines: *F*_A_; dotted burgundy lines: *F*_B_; black solid line: *A*_1tot_=*A*_1A_+*A*_1B_) for the exception of the “mutated” *A*_1A_ kinetics which are shown in pink. The grey dotted lines show the simulations of *A*_1tot_ relaxation in the wild-type for ease of comparison.

**Figure S4.** Energetics (A, C, E) and kinetic simulations (B, D, F) of the electron transfer reactions downstream of *A*_1_ in the three classes of mutants of the PsaA reaction centre subunit, starting from the free energy for = – 50 meV and = –220 meV in the wild-type scenario (Milanovsky et al. 2017). **A** and **B** simulate the “moderately speed up” scenario, ($\Delta\Delta G_{A_{1\text{A}}}^{0}$= – 25 meV); **C** and **D** the “moderately slowing down” scenario ($\Delta\Delta G_{A_{1\text{A}}}^{0}$= + 35 meV); **E** and **F** the “extreme slow-down” scenario ($\Delta\Delta G_{A_{1\text{A}}}^{0}$= + 110 meV). In all energetics panels the shifted redox midpoint potential induced by the specific mutation is indicated in pink, whereas the wild-type potential is shown as grey dotted lines. In the kinetic simulations the same colour-coding as in Figure 3, S1 and S2 is used (dash-dotted blue lines: *A*_1B_; dotted golden lines: *F*_X_; dotted orange lines: *F*_A_; dotted burgundy lines: *F*_B_; black solid line: *A*_1tot_=*A*_1A_+*A*_1B_) for the exception of the “mutated” *A*_1A_ kinetics which are shown in pink. The grey dotted lines show the simulations of *A*_1tot_ relaxation in the wild-type for ease of comparison

**REFERENCES**

Ptushenko, V.V., Cherepanov, D.A., Krishtalik, L.I., Semenov, A.Y. (2008). Semi-continuum electrostatic calculations of redox potentials in photosystem I. *Photosynth. Res.* 97, 55–74. doi: 10.1007/s11120-008-9309-y

Milanovsky, G.E., Petrova, A.A., Cherepanov, D.A, Semenov A.Y. (2017) Kinetic modeling of electron transfer reactions in photosystem I complexes of various structures with substituted quinone acceptors. *Photosynth. Res.* 133: 185–199. doi: 10.1007/s11120-017-0366-y
